# Choosing the Appropriate Target for the Treatment of Psoriatic Arthritis: TNFα, IL-17, IL-23 or JAK Inhibitors?

**DOI:** 10.31138/mjr.33.1.150

**Published:** 2022-04-15

**Authors:** Chrysoula G. Gialouri, Gerasimos Evangelatos, George E. Fragoulis

**Affiliations:** 1 First Department of Propaedeutic Internal Medicine, Medical School, National and Kapodistrian University of Athens,“Laiko” General Hospital, Athens, Greece

**Keywords:** psoriatic arthritis, biologic DMARDs, JAK-inhibitors, treatment, comorbidities

## Abstract

Psoriatic arthritis (PsA) is a highly heterogenous disease. Apart from arthritis and psoriasis, other manifestations can also occur, including enthesitis, dactylitis, axial-, nail-, eye- and bowel- involvement. Comorbidities are also frequent in the setting of PsA, with cardiovascular disease and mental-health disorders being the most frequent. The Rheumatologist’s arsenal has many different treatment options for treating PsA. Despite their effectiveness, there are some differences in terms of efficacy and safety that might affect clinician’s decision for one or the other drug. Comparing biologic DMARDs and JAK-inhibitors, one could say that they have similar effectiveness in terms of musculoskeletal manifestations. However, anti-IL-17 and anti-IL-23 drugs seem to be more effective for skin manifestations. In contrast, JAK-inhibitors and etanercept might be less effective for these manifestations. Inflammatory bowel disease and uveitis are non-responsive to etanercept and anti-IL-17 drugs. As regards to comorbidities, data are scarce, but future studies will shed light on possible differential effect of bDMARDs or JAK-inhibitors. Safety is always an important drive for choosing the appropriate treatment. Infections are the most common adverse event of these drugs. Etanercept and anti-IL-17 drugs are safer for patients having latent tuberculosis, while herpes zoster is more common in individuals receiving JAK-inhibitors. Finally, venous thromboembolism risk, should be taken into account when JAK-inhibitors are used. In this review, we comparatively present, as outlined above, the various aspects that could affect the choice of the appropriate bDMARD or JAK-inhibitor for the treatment of a PsA patient.

## INTRODUCTION

Psoriatic Arthritis (PsA) is an inflammatory arthritis classified into the group of spondyloarthritides (SpA), affecting approximately one-third of patients with psoriasis. In the general population, the prevalence of PsA ranges from 0.1% to 0.25%.^1^ It is a multifaceted disease, including peripheral arthritis, axial involvement with sacroiliitis and/or spondylitis, enthesitis, dactylitis, skin and nail lesions, while extra-articular manifestations such as uveitis and inflammatory bowel disease (IBD) are not rare through-out disease course.^2^

PsA is often accompanied by other clinical conditions. Cardiometabolic comorbidities such as obesity, type 2 diabetes mellitus (DM), non-alcoholic fatty liver disease (NAFLD), dyslipidemia, and hypertension are prominent.^3^ In fact, a recent meta-analysis, has shown that about 30% of PsA patients meet the diagnosis for metabolic syndrome (MetS).^4^ Also, depression is a comorbidity frequently observed with a prevalence ranging between 15% and 20%.^2^

Pathophysiology of PsA is based on a complex interplay between environmental stimuli (infections, trauma, smoking, stress etc.), gut microbiome and mechanical stress, in genetically predisposed individuals.^5^ Upon this background, myeloid dendritic cells activated by interferon (IFN)-a and other pro-inflammatory mediators produced by innate immune cells (plasmacytoid dendritic cells and natural killer T cells, macrophages, keratinocytes), drive through IL-12 and IL-23 to Th1 and Th17 responses, respectively. The former lead to production of TNF-a, IFN-γ and the latter of IL-17, IL-22.^6^ Subsequently, these cytokines mediate their effect in a variety of cells, like resident skin cells, synovial tissue cells, osteoblasts and osteoclasts, leading to disease initiation and/or perpetuations.^5^

There are several therapeutic options in PsA, including NSAIDs, glucocorticoids, conventional disease modifying antirheumatic drugs (cDMARDs; methotrexate, sulfasalazine, leflunomide, cyclosporin), targeted synthetic (ts) DMARDs [JAK-inhibitors (JAKi) and apremilast] and biologic DMARDs (bDMARDs), including regimes against TNF, Interleukin (IL)-17 and IL-23.

Treatment, especially with bDMARDs, in patients with PsA has come with some concerns for safety, including risk for malignancies, infections and cardiovascular events. Choice of the drug is based on several features, like efficacy in the various facets of PsA, comorbidities, contraindications (eg, NSAIDs and gastric ulcers), safety profile (eg, some biologics are safer for TB than the others) and other aspects like route of administration. Aim of our review is to describe how the clinician chooses the appropriate treatment, among bDMARDs and JAKi (also known as JAKinibs), based on the above-mentioned axes.

## COMPARISON OF EFFICACY

**Figure 1.**
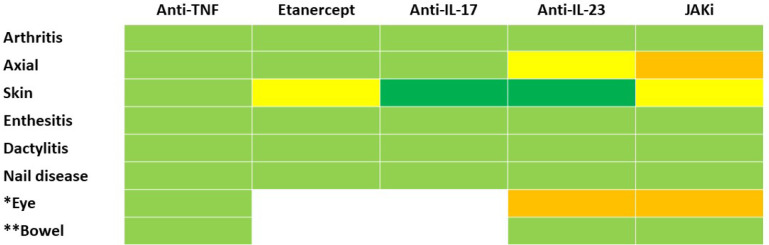
Efficacy of the various treatment modalities in various aspects of PsA. Green: efficacy, dark green: category is superior compared to the others, white: inefficacy or contraindication, orange: trials are underway, yellow: limited efficacy or use with caution. *Marketed for uveitis: only adalimumab; **Marketed for Crohn’s disease: adalimumab, infliximab, certolizumab, ustekinumab. Marketed for ulcerative colitis: adalimumab, infliximab, golimumab, ustekinumab, tofacitinib. VTE: venous thromboembolism; PE: pulmonary emboli; JAKi: JAK inhibitors.

Evidence from clinical trials and observational studies have revealed differences regarding the efficacy of bDMARDs and JAKi in various aspects of PsA (**Figure 1**).

### Peripheral Arthritis

European Alliance of Associations for Rheumatology (EULAR), American College of Rheumatology (ACR) and Group for Research and Assessment of Psoriasis and Psoriatic Arthritis (GRAPPA) guidelines suggest that treatment with a cDMARD should be initiated in most PsA patients with active peripheral arthritis.^7–9^ For those who have inadequate response or cannot tolerate cDMARDs, a bDMARD should be commenced. Regarding articular outcomes, all TNF-inhibitors (TNFi), IL-17 inhibitors, ustekinumab (an IL-12/23 inhibitor), guselkumab (an IL-23 inhibitor) and tofacitinib (a JAK1-3 inhibitor) have been proven effective in clinical trials. In a recent meta-analysis, TNFi, IL-17 inhibitors and ustekinumab had all increased ACR20, ACR50 and ACR70 responses versus placebo, with comparable risk ratios.^10^ Secukinumab exhibited similar efficacy with adalimumab in arthritis outcomes in EXCEED study, a head-to-head double-blind randomised control trial.^11^ As for ixekizumab, another IL-17 inhibitor, it showed numerically similar results to adalimumab in bDMARD-naïve patients with active PsA in SPIRIT-P1 study.^12^ To be mentioned, the latter study was not powered to directly compare the two drugs.^10,12^ Ustekinumab initially demonstrated numerically lower efficacy than other bDMARDs, by indirect comparison, as measured by ACR20, ACR50 and ACR70 in pivotal studies PSUMMIT-1 and PSUMMIT-2.^7,13^ However, in ECLIPSA, a small randomized open-label study, ustekinumab was compared to TNFi (specifically adalimumab, certolizumab, etanercept and infliximab) and showed similar effect on arthritis (p=0.95).^14^ Collectively, TNFi and inhibitors of IL-17 and IL-12/23 (secukinumab, ixekizumab, ustekinumab) had similar ACR20 responses (risk ratio for TNFi 2.23, 95% CI 1.60–3.11 and pooled risk ratio for non-TNFi 2.30, 95% CI 1.94–2.72).^15^

As for tofacitinib, its articular effectiveness is comparable to most bDMARDs.^16^ However, a recent network meta-analysis showed that in TNF-naïve patients, etanercept, golimumab and infliximab seem to achieve better ACR20 responses compared to tofacitinib, while in TNF-inadequate responders (TNF-IR) PsA patients, certolizumab has been found to perform better than tofacitinib in peripheral arthritis management.^16^ Guselkumab was recently approved by FDA for the treatment of active PsA. In pivotal studies DISCOVER-1 and DISCOVER-2, guselkumab exhibited ACR20 response in 52–64% of patients with active PsA at 24 weeks,^17,18^ but no direct comparison with other drugs is available till now. Other IL-23 inhibitors, such as risankizumab and tildrakizumab, are currently under investigation for the treatment of active PsA.

Finally, all TNFi, IL-17 inhibitors, ustekinumab and tofacitinib are associated with improvement of physical functioning in PsA patients, as assessed by health assessment questionnaire-disability index (HAQ-DI).^16^ TNFi did not differ significantly from newer agents (inhibitors of IL-17 and IL-12/23) in this parameter (pooled risk ratio of 0.29 [95% CI −0.39 to −0.19] versus −0.26 [95% CI −0.31 to −0.22]).^15^ To be mentioned, in TNF-naïve patients, secukinumab 150mg/4weeks did not lead to significant change of HAQ-DI from baseline.^16^ Finally, numerically comparable reduction in HAQ-DI values was reported with ixekizumab and adalimumab in SPIRIT-P1 study.^12^

### Axial disease

Scarce data are available for bDMARD effectiveness in psoriatic spondylitis. MAXIMISE is the only clinical trial with focus on axial disease in patients with PsA. In this study, both secukinumab doses 150mg and 300mg/4 weeks, after loading dose, achieved statistically significant ASAS20 response compared to placebo (19). In patients with PsA, treatment with etanercept led to improvement in Bath Ankylosing Spondylitis Disease Activity Index (BASDAI) and Bath Ankylosing Spondylitis Disease Functional Index (BASFI) scores.^20^ Based on data from Axial SpA (AxSpA), EULAR and ACR suggest that PsA patients with axial disease should start a TNFi after NSAIDs failure; an IL-17 inhibitor is the suggested alternative if there are contraindications for TNFi or severe skin involvement.^7,8^ Noteworthy, blockade of IL-23 is not effective in AxSpA, as indicated from negative results from ustekinumab and guselkumab trials.^13,21^ However, it still remains to be defined whether results regarding therapeutic efficacy are transferable from AxSpA to axial-PsA. Finally, several JAKinibs are currently under investigation for the treatment of AxSpA, with promising initial results. Tofacitinib achieved significantly higher (Assessment of SpondyloArthritis-20) ASAS20 and ASAS40 rates compared to placebo in a phase II trial in bDMARD-naïve patients with active ankylosing spondylitis.^22^

### Psoriasis

All TNFi, IL-17 inhibitors, IL-12/IL-23 and IL-23 inhibitors are approved for plaque psoriasis.^23^ As shown in a recent meta-analysis, Psoriasis Area Severity Index (PASI) 75 and PASI90 responses were comparable between TNFi (considered as a class), IL-17 inhibitors and ustekinumab in patients with PsA.^10^ Another meta-analysis showed that IL-17 inhibitors, IL-23 inhibitors and infliximab are associated with increased rates of PASI90, compared to ustekinumab and other TNFi.^24^ Importantly, etanercept has shown lower rates of PASI75 response than the rest TNFi,^25^ while adalimumab proved better than certolizumab in achieving PASI90 response.^24^ Moreover, etanercept was proven inferior to ustekinumab,^26^ secukinumab,^27^ and ixekizumab^28^ in psoriasis. Ustekinumab performed better than TNFi in psoriatic skin disease (p=0.03) in ECLIPSA study.^14^ Newer IL-23 inhibitors, especially guselkumab and rizankizumab, have achieved impressive PASI75 and PASI90 scores, even better than ustekinumab, in clinical trials.^13^ As expected, guselkumab was superior to adalimumab in a head-to-head comparison in psoriasis patients.^29^ Taking the above data into account, IL-17 and IL-12/23 inhibitors are preferred over TNFi in PsA patients with severe skin disease, according to EULAR guidelines.^7^

Tofacitinib, on the other hand, seems inferior to bDMARDs in skin manifestations of PsA,^24^ especially in the dose of 5mg BID. Based on a recently published meta-analysis, golimumab, infliximab and ixekizumab were associated with increased PASI75 response compared to tofacitinib in TNFi-naïve patients.^16^ In TNF-IR patients, tofacitinib 5mg BID did not differ significantly from placebo in PASI75 rates.^16^

### Enthesitis

Enthesitis has been characterised as a hallmark of PsA, occurring in about 35–50% of patients. Enthesitis in clinical trials is usually quantified with Maastricht Ankylosing Spondylitis Enthesitis Score (MASES) index or Leeds Enthesitis Index (LEI). NSAIDs are widely used in active enthesitis in clinical practice, but are less effective in chronic enthesitis.^30^ As cDMARD have been proven ineffective in the treatment of enthesitis,^30^ EULAR, ACR and GRAPPA guidelines suggest the initiation of a bDMARD in patients with active enthesitis (7–9).

All TNFi, IL-17 inhibitors secukinumab and ixekizumab, ustekinumab and tofacitinib have exhibited satisfactory results in PsA enthesitis.^10,15,30,31^ ECLIPSA study was the only study with enthesitis resolution as the primary outcome.^14^ In this open-label randomised controlled study, in about 74% of ustekinumab-treated patients and 41.7% of patients treated with a TNFi complete clearance of enthesitis was noted at 24 weeks (p=0.018).^14^ In contrary, a recent meta-analysis showed comparable mean risk ratios for enthesitis resolution between IL-17 inhibitors, TNFi and ustekinumab, compared to placebo (2.31, 1.99 and 1.41, respectively).^10^ Moreover, in another meta-analysis, TNFi demonstrated similar rates of enthesitis resolution at week 24, compared to IL-17 and IL12/23 inhibitors.^15^ In EXCEED, a head-to-head clinical trial of secukinumab versus adalimumab in PsA, both drugs had similar rates of enthesitis remission, as defined by LEI (61% vs 54%, p=0.150).^11^ Moreover, ixekizumab showed numerically greater enthesitis improvement versus adalimumab, especially in the dose of 80mg every 2 weeks, in the SPIRIT-P1 study.^12^ Tofacitinib in the dose of 5mg twice daily had numerically lower improvement in LEI compared to adalimumab at 3 months, but the results of the two drugs were comparable at month 12.^31^ Thus, tofacitinib in the approved dose of 5mg BID might have a more delayed effect on enthesitis than adalimumab. Finally, in clinical trials, guselkumab led in enthesitis resolution in 40–50% at 24 weeks, depending on the dose applied (17, 18). Similar or even better results were reported on 56 weeks in a phase II trial.^32^

### Dactylitis

Dactylitis is a characteristic manifestation of PsA and occurs in about half patients during the course of the disease.^33^ Although no controlled studies have been conducted regarding the efficacy of NSAIDs and local corticosteroids in dactylitis,^33^ these agents are frequently used by clinicians. As digital inflammation most times accompanies a generally active articular disease, EULAR and GRAPPA suggest that initiation of a cDMARD, mainly methotrexate, should be considered.^7,9^

Monoclonal antibody TNFi (certolizumab, infliximab, golimumab and adalimumab), IL-17 inhibitors, ustekinumab and tofacitinib have been proven effective on dactylitis in clinical trials.^16,33^ GO-DACT was the only trial that a dactylitis score change was the primary outcome.^34^ This trial demonstrated that the combination of golimumab plus methotrexate exhibited greater improvement of dactylitis, compared to methotrexate monotherapy, in methotrexate- and bDMARD-naïve patients.^34^ Although in clinical trials of ustekinumab a favourable effect on dactylitis was shown,^35^ a recent meta-analysis showed that the reduction of dactylitis was not statistical significant.^10^ On the other hand, in the same meta-analysis, IL-17 inhibitors and TNFi were both proved effective in dactylitis resolution (2.65 and 2.07 risk ratio versus placebo, respectively).^10^ It seems that TNFi and IL-17/IL-12/23 inhibitors have comparable efficacy in dactylitis.^15^ In EXCEED study, secukinumab and adalimumab did not differ significantly in dactylitis resolution rates (75% vs 70%, p=0.356).^11^ In addition, in SPIRIT-P1 study, ixekizumab and adalimumab showed similar rates of dactylitis amelioration.^12^ Finally, tofacitinib and adalimumab have also comparable results in Dactylitis Severity Score change in cDMARD-IR patients.^16,31^ To be noted, guselkumab was also proved effective in dactylitis; in guselkumab-treated PsA patients with dactylitis at baseline, 59–65% had remission of digital inflammation at 24 weeks.^17,18^ These results improved further at 56 weeks.^32^

### Nail involvement

About 50% of psoriasis patients and 80% of patients with PsA have nail lesions.^36^ Nail Psoriasis Severity Index (NAPSI) has been utilized in most studies to quantify the extent of nail psoriasis. All TNFi, IL-17 inhibitors, ustekinumab, guselkumab, and tofacitinib have shown effectiveness in nail disease. EXPRESS study showed significant reduction in NAPSI score at weeks 10 and 24 of treatment with infliximab in patients with psoriasis.^37^ Data from psoriasis trials suggest that etanercept is effective in nail psoriasis.^38^ Adalimumab, golimumab and certolizumab have been proven effective in treating nail involvement in patients with PsA.^12,38–40^ Ustekinumab improves nail-disease in patients with moderate-to-severe psoriasis in 24 weeks and the improvement continued until week 52 of treatment.^41^ Sustained and strong improvement of nail psoriasis has been recently reported with secukinumab treatment.^42^ Moreover, significant NAPSI score improvement was reported in ixekizumab-treated PsA patients, numerically comparable to adalimumab.^12^ Importantly, guselkumab was also proven very effective in nail psoriasis, without significant difference from adalimumab.^43^ Finally, improvement in nail psoriasis has been reported with 16 weeks tofacitinib treatment and the result was maintained for at least 52 weeks.^44^

### Inflammatory bowel disease

About 3.3% of PsA cases might express clinically evident IBD development,^45^ while subclinical intestinal inflammation can be detected in up to 40% of PsA patients.^46^ Only few data have been published regarding effectiveness of available drugs in PsA patients with concurrent IBD (**Figure 1**). Thus, management of these patients is based on data from IBD trials. Monoclonal antibody TNFi and ustekinumab are approved for the treatment of Crohn’s disease and ulcerative colitis.^23^ In 70 patients with IBD and psoriasis or PsA, the majority of patients achieved clinical remission of intestinal, skin and articular manifestations with ustekinumab.^47^ Etanercept and inhibitors of IL-17 have been proven ineffective in the treatment of IBD.^23,48^ On this basis, ACR recommends that PsA patients with concomitant IBD are preferably treated with a monoclonal antibody TNFi or ustekinumab.^8^ Nevertheless, the risk of IBD flare or new-onset IBD in patients treated with secukinumab is low.^49^ Regarding tofacitinib, it has been approved for active ulcerative colitis, so this makes tofacitinib a useful alternative in patients with PsA and ulcerative colitis. In contrary, tofacitinib has not been effective in Crohn’s disease.^50^ Finally, ongoing phase III clinical trials will examine the efficacy of guselkumab in Crohn’s disease and ulcerative colitis.

### Eye involvement

From ocular manifestations of PsA, anterior uveitis is the most common and can affect up to 25% of PsA patients.^51^ Data on management of specific PsA-associated uveitis are lacking, thus, treatment modalities used for SpA-associated uveitis are applied. Adalimumab has been approved for non-infectious uveitis (**Figure 1**) and, along with infliximab, are the most potent bDMARDs in the treatment of SpA-associated uveitis.^52^ Certolizumab and golimumab have shown promising results in reducing uveitis flares;^53^ in contrary, etanercept was ineffective in uveitis (compared to adalimumab and infliximab) and might be associated with a slightly increased incidence.^52^ In a pooled analysis of 118 patients with non-infectious uveitis, secukinumab failed to reduce recurrence of ocular inflammation, but contributed in immunosuppressants use reduction.^54^ Ustekinumab and tofacitinib showed promising results in case reports and are currently under study in ongoing clinical trials.^52^ Ixekizumab and guselkumab have not been studied yet in non-infectious uveitis.^53^ Based on the aforementioned data, EULAR recommendations suggest that PsA patients with uveitis are treated with a monoclonal antibody against TNF, as a first- and second-line treatment.^7^ Moreover, as uveitis can respond to methotrexate, ACR recommends the use of combination of bDMARD with methotrexate instead of bDMARD monotherapy in patients with PsA-associated uveitis.^8^

## COMORBIDITIES IN PSA: EFFICACY OF bDMARDS AND JAK-INHIBITORS

### Cardiometabolic comorbidities and associated factors: obesity, DM, NAFLD, dyslipidemia, cardiovascular diseases

As mentioned above metabolic comorbidities and associated risk factors (eg, obesity, impaired glucose tolerance, dyslipidemia) are commonly encountered in PsA and strongly linked with morbidity and mortality in this setting.^3^

#### Obesity

Obesity is identified as an independent risk factor for PsA development in patients with psoriasis, as well as in healthy individuals.^55^ Additionally, adipose tissue and its mediators (ie, adipokines) seem to contribute to the perpetuation of inflammation in these patients. There is some evidence that anti-TNF treatment might result in weight gain, however it is not clear whether it concerns an increase in fat or free-fat mass.^56,57^ On the other hand, obesity is poor predictor for treatment response^58,59^; Treatment with TNFi is found to be less effective for achieving and sustaining remission.^58,60^ A recent study by Ogdie et al revealed that among other factors, obesity was negatively associated with disease remission in patients starting TNFi (OR = 0.51, 95% CI 0.32–0.81).^61^ Furthermore, cohort studies based on Danish and Icelandic biologics registries including over 1000 PsA patients pointed out that obesity was a risk factor for TNFi withdrawal owing to reduced response.^62^ Data for other biologics in obese PsA patients are limited. There are findings indicating that Th17 cells and IL-17 play some role in the obesity-related inflammatory processes.^63^ Pantano et al. prospectively analysed 100 PsA patients receiving secukinumab for 6 months and found that overweight/obese patients (BMI≥ 25) had better clinical response (estimated using Disease Activity in Psoriatic Arthritis [DAPSA] score) than those with BMI<25 (p=0.05).^64^ In contrast, results from a retrospective study in PsO patients show inferior efficacy of sekucinumab in those with BMI ≥ 30.^65^

#### Diabetes Mellitus

DM is more prevalent among PsA patients, especially in women with more active disease, compared with the general population. The pathogenic linkage between PsA and DM is multifactorial. Among other cytokines, TNF-a plays a critical role leading to insulin resistance (IR) and higher levels of active endogenous cortisol, affecting in turn, glycose metabolism. Also, TNF-a downregulates adipokines which normally increase insulin sensitivity and have anti-atherogenic properties.^66^ Also, type 2 DM has been correlated with increased circulating Th17 cells and IL-17 levels,^67^ while the pleomorphic actions of JAKi do not allow any definite conclusions about their impact in glucose homeostasis. Although there is some evidence that TNFi might have some beneficial effect, it appears that treatment with bDMARDs, does not significantly alter glucose homeostasis. A prospective study, including PsA patients without DM, demonstrated that treatment with TNFi (adalimumab, infliximab and etanercept) up to 6 months, did not change fasting glucose levels (FGL).^68^ On the other hand, commencing TNFi in patients with inflammatory arthritis has been shown to lead in reduc -tions in HbA1c. This effect, however, had comparable magnitude with patients receiving methotrexate (MTX), implying that this reduction might not be TNFi-specific.^69^ As regards IL-17-blocking reagents, thus far, large-scale studies including PsA/PsO patients receiving monoclonal antibodies against IL-17A (ixekizumab, secukinumab) have not found any effect on glucose metabolism.^70–73^ Finally, data derived from two phase 3 studies (OPAL Broaden and OPAL Beyond) in PsA patients receiving JAKi, indicate that, irrespective of baseline metabolic state, tofacitinib was effective for patients with active PsA.^74^

#### Non-alcoholic fatty liver disease

NAFLD comprises a spectrum of liver disease ranging from hepatic steatosis, steatohepatitis and liver fibrosis (LF) to cirrhosis and potentially carcinoma.^75^ In a meta-analysis the risk of NAFLD compared to non-psoriatic controls was elevated in patients with psoriasis and more pronounced in PsA patients (OR: 2.25, 95% IC: 1.37–3.71).^76^ Besides, insulin resistance and MetS are predisposing factors for NAFLD occurrence.^77^ It is not clear whether liver disease in PsA is promoted by the treatment administered -especially with methotrexate- or is associated with disease itself.^58^ Seitz et al. showed that the combination of TNFi with methotrexate (MTX) has a protective effect against development of LF in PsA patients compared to MTX monotherapy,^78^ while in a retrospective study, using data derived from claims database, TNFi use was not associated with a protective effect for PsA patients.^79^ Hitherto, there are no data about the possible role of IL-17 inhibitors or other bDMARDs in NAFLD/hepatic steatosis, although there are some data supporting that Th-17 and IL-17 promote hepatic steatosis and inflammation in NAFLD patients.^80^

#### Dyslipidemia

Dyslipidemia is also a common feature in PsA. However, the abnormalities in the lipid profiles in these patients are ill-defined. Findings from a limited number of studies support that PsA patients, display lower levels of TC, HDL-C and LDL-C, but higher levels of TG compared to individuals without PsA.^81^ It is also unknown if the lipid paradox described in RA,^82^ operates also in PsA. Studies about the effect of bDMARDs in lipid profile of PsA patients are lacking. In a cohort study including 118 PsA patients, 5-years treatment with etanercept led to a modest increase of TC, HDL-C and LDL-C, TC/HDL-C ratio remained unchanged, whereas ApoB/ApoA-I ratio decreased implying thus a cardioprotective effect.^83^

### Cardiovascular Disease

It is well recognized that cardiovascular risk is increased in patients with inflammatory arthritis.^81,84^ Many hypotheses have been formed to explain it; however, chronic inflammation appears to be the main culprit.

In particular for PsA, available data from a meta-analysis indicate that it is associated with increased risk of cardiovascular disease (CVD) and 55% higher risk of developing an incident cardiovascular event (myocardial infarction [MI], cerebrovascular diseases and heart failure [HF]), compared with the general population.^85^ The higher cardiovascular risk cannot be fully explained by the traditional cardiovascular risk factors and chronic systemic inflammation seems to contribute. To that end, role of classical pro-inflammatory cytokines like IL-6 and TNF is better recognized, while data about IL-17 and/or IL-23 and atherosclerosis are still contradictory.^3,86^ It seems that except from controlling traditional cardiovascular risk factors, amelioration of systemic inflammation by bDMARD treatment plays important role in reducing cardiovascular risk in these patients.

Di Minno et al. documented an important reduction of carotid intima-media thickness (cIMT) and lower number of carotid plaques, both used as surrogate markers of atherosclerosis, in PsA patients treated with TNFi, in contrast to those receiving cDMARDs. In addition, treatment duration with TNFi was inversely correlated with cIMT progression, supporting the concept of accumulating anti-inflammatory impact of TNFi treatment on vascular lesions.^87^ In concert, Eder et al. in two cohort studies ascertained firstly that TNFi reduced the deterioration of carotid plaques, only in males. Secondly, after one year of TNFi therapy, vascular inflammation was found to be improved in both genders, regardless of traditional cardiovascular risk factors.^88^ The beneficial vascular effects of anti-cytokine immune therapy is correlated with improved clinical outcomes. Although data are more robust for other inflammatory arthritis, like RA, for PsA only a few studies have evaluated the effect of bDMARDs on CVD risk, let alone the potential differences among different bDMARDs and JAKinibs. A meta-analysis including only 6 studies for PsO/PsA, shown that TNFi therapy is associated with lower risk of all CVD, than the topical treatment.^89^ In line with these findings, another meta-analysis shown remarkable reduction of MI incidence and risk of CVD with TNFi, compared with topical therapy, phototherapy or methotrexate treatment (90). On the other hand, when other licensed bDMARs are compared with TNFi, no differences were found. In a large cohort study including 60028 patients with PsO or PsA from US, the risk for atrial fibrillation or major adverse CVD events did not diverge between groups treated with ustekinumab (IL-12/IL-23 inhibitor) or TNFi.^91^ As for JAK-inhibitors (JAKi), short-term safety data from OPAL Balance clinical trial, including > 650 PsA patients, do not indicate increased risk for major adverse cardiovascular events comparable to other treatment groups.^92^

### Mental Health Disorders

Anxiety and depression are frequent comorbidities in the setting of PsA^2^ and have been linked with worse clinical outcomes and lower probability of achieving disease remission.^93^ Although they can be owed to devastating clinical symptomatology, pain and reduced quality of life, they are mechanistically linked with inflammatory processes and mediators like IL-6 and TNF. Data are not robust for PsA, but it seems that treatment with bDMARDs has beneficial effect also on concomitant mental health disorders. Kappelmann et al. in a meta-analysis showed that adalimumab, etanercept, infliximab and tocilizumab had beneficial effect in depressive symptomatology, in a variety of immune-mediated diseases.^94^ Interestingly, in a recent mega-analysis it was found that IL-12/-23 and IL-6 blockers demonstrated larger effects on depression occurring in immune-mediated diseases, compared to other bDMARDs.^95^

## SAFETY

### Infections

Infections are probably the most well recognised concern about the use of b- and ts-DMARDs. Overall, there are no differences for serious infections across bDMARDs and JAKinibs.^96^ However, a recent interim analysis of a still ongoing study (A3921133) comparing tofacitinib with adalimumab showed that serious infections were increased for JAKi in individuals aged more than 65 years old. Subsequently, EMA recommended that tofacitinib should be used in this subgroup of patients only when there is no other alternative.^97^ Another study analysing data from RA RCTs and CORRONA registry showed that serious infections for patients treated with tofacitinib was similar to adalimumab for 5mg twice a day (bid) dosing scheme, but higher than adalimumab for 10mg bid.^98^ More data will be accumulated over the next years. However, 10mg bid should be avoided in people aged over 65 years or those with an increased risk for infections.

Finally, some regimes are safer than others regarding specific infections, as outlined below.

#### Tuberculosis

Tuberculosis (TB) has been identified as the most common opportunistic infection among patients with autoimmune rheumatic diseases. Actually, it is well known that latent TB reactivation or *de novo* cases of TB are associated with TNFi treatment^99,100^ offering a 4–8 times higher risk. This is further increased in endemic regions for TB.^101^ This association could be explained having in mind the essential role of TNF-a and IFN-γ for immune cells’ recruitment, phagocytosis of mycobacteria and granuloma formation.^102,103^ Of note, therapy with soluble TNF receptor (etanercept) is less likely to cause TB compared to anti-TNF monoclonal antibody (mAb) agents.^104^ Other cytokines, like interferons, IL-12, IL-17, IL-22 (105) are also implicated in immune response to mycobacterial infection. However, available data derived from clinical trials and post-marketing surveillance for IL-17-targeted agents in PsA and PsO patients suggest that the risk for TB infection/reactivation, upon treatment with these regimes is not high.^106–108^ Furthermore, despite the protective role of IL-12 and IL-23 against mycobacterium tuberculosis,^109^ no cases of active TB have been reported in PsA patients treated with ustekinumab,^110,111^ neither with selective anti-IL-23 mAbs.^112^ Finally, JAK proteins intervene in IL-12/IL-23 and IFN-γ signalling^113^ and mutations in relevant genes are deemed to be responsible for vulnerability to mycobacterial infections.^114^ Studies in RA patients indicate that the incidence of TB with tofacitinib is comparable to what observed with TNFi.^115^ Moreover, the risk seems to parallel with higher drug doses and depends on the regional prevalence of TB. As for PsA patients, data are limited and short-term safety results from clinical trials do not report cases of TB under JAKi therapy.^116^

**Figure 2:**
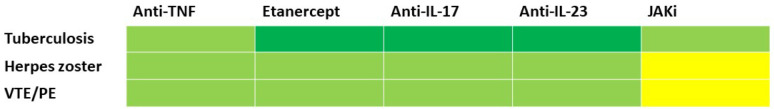
Adverse events of bDMARDs and JAKi in PsA. Green: safe (taking into account all necessary screening procedures and prophylaxis); dark green: better safety profile compared to the others; yellow: use with caution.

To sum up, although some regimes (eg, etanercept or IL-17/IL-23 inhibitors) might be safer than others (**Figure 2**), in everyday clinical practice, screening for latent TB should be recommended for all patients before initiating bDMARDs and JAKi.^117^

#### Herpes Zoster

Herpes zoster (HZ) primary infection or reactivation has been reported as an adverse event closely linked with JAKi. The underlying pathogenetic mechanisms for this are not entirely clear. However, we know that JAKSTAT pathways are integral parts of adaptive immune response to intracellular pathogens, like viruses and that JAK family proteins are involved in many steps of this virus life cycle.^118^ It is clear that the risk of HZ reactivation with JAKi is higher compared to bDMARDs (**Figure 2**). A real-world study in RA patients found approximately double incidence rate (IR) of HZ with tofacitinib compared to TNFi, abatacept, rituximab and tocilizumab.

HZ reactivation in this context is mild, being usually, but not always, limited to a single dermatome.^118^ Risk factors augmenting the HZ reactivation risk include female sex, age ≥65 years, concomitant or previous corticosteroid therapy (prednisolone >7.5mg per day), tofacitinib dose and Asian ancestry.^119–121^ Additionally, the risk seems to be lower in patients receiving tofacitinib-monotherapy, compared to those treated with combination therapy with cDMARDs.^122^ Finally, it is still debatable, whether some JAKi are safer than the others in terms of HZ reactivation, although this association seems to be a class effect. The risk seems to be comparable between RA and PsA patients.^92^ In conclusion, JAKi should be avoided in patients who have a past medical history of HZ infections, while it is unclear whether re-introduction of therapy with JAKi is a reasonable option after HZ reactivation.

#### Fungal infections

Risk for candidiasis is increased in patients receiving anti-IL-17 reagents, resulting in adjusted incidence rates of 0.4–2.2/100 patient-years.^106,123^ This is probably due to the central role of IL-17 in the defence against fungal infections.^124^ Of note, candidiasis in this setting is usually mild and does not lead to treatment discontinuation.

### Malignancies

Malignancy rates in PsA receiving treatment with bDMARDs or JAKi seem to be similar to what observed in the general population, except from non-melanoma skin cancer (NMSC), which prevalence has been found to be increased. There are not observed differences across different drug categories and screening strategies are not yet defined in patients receiving bDMARDs/tsDMARDs.^117,125–128^

### Venous Thromboembolism

Venous thromboembolism (VTE) and pulmonary embolisms (PE) are two adverse events that have been described in the context of treatment with JAKi (**Figure 2**). So far, data are more solid for tofacitinib, for which EMA recommended that should not be used at the 10mg bid dose for ulcerative colitis, unless there is no other option. Newer data from an interim analysis of open label trial (A3921133 study) of RA patients older than 50 years old, showed that the risk for PE was 3 and 6 times higher for tofacitinib 5 and 10mg bid, respectively.^97^ This has led EMA to recommend that tofacitinib should be used with caution for all dosing schemes and indications, when risk factors for cardiovascular or thromboembolic disease (eg, obesity, diabetes, prolonged immobility) concur. For baricitinib, data are less robust with VTEs being numerically higher in studies assessing the efficacy of this drug.^129,130^ Food and Drug administration (FDA), has approved only the lower (2mg/day) dosing scheme for rheumatoid arthritis. For other JAKi, more data are needed before we can draw a conclusion whether VTE/PE is a class effect.

### Heart failure (HF)

Although biologics offer benefit in terms of cardiovascular outcomes, including myocardial infarction and cardiovascular events, severe heart failure (NYHA class III and IV) is a relative contraindication for treatment with TNFi.^131^ In a recent meta-analysis investigating the effects of various medications used in inflammatory arthritis in cardiovascular outcomes, no effect of TNFi was found on occurrence of heart failure.^89^ As the authors state though, this could be owed in a selection bias, as clinicians would avoid these regimes in patients with heart failure.

## CONCLUSION

In conclusion, there are several features that can affect clinician’s decision for one or the other bDMARD. Anti-IL-17 and anti-IL-23 are better than other bDMARDs for patients with severe psoriasis, while for arthritis, enthesitis and dactylitis, no major differences are noted. IL-17 blockers should be avoided for IBD, while TNFi (except for etanercept) seem to be the better option, so far, for eye involvement. For comorbidities, evidence is still scarce, but future studies might show some benefit for some of the drugs used for PsA treatment. Safety is always a drive for choosing the appropriate treatment. Etanercept, anti-IL-17 and anti-IL-23 seem to be safer regarding TB, while HZ as well as VTE/PE should be taken into account when JAKi are prescribed. Apparently, this clinically oriented review does not disregard the phenotypic variety of PsA. Data from studies using newer technologies (eg, omics) will help to better identify subgroups within PsA and thus, guide tailor treatment approach.

## References

[1] Ogdie A Weiss P. The Epidemiology of Psoriatic Arthritis. Rheum Dis Clin North Am 2015;41(4):545–68. 26476218 10.1016/j.rdc.2015.07.001 PMC4610151

[2] Fragoulis GE Evangelatos G Tentolouris N Fragkiadaki K Panopoulos S Konstantonis G Higher depression rates and similar cardiovascular comorbidity in psoriatic arthritis compared with rheumatoid arthritis and diabetes mellitus. Ther Adv Musculoskelet Dis 2020;12:1759720X20976975. 10.1177/1759720X20976975 PMC7727079 33343726

[3] Ferguson LD Siebert S McInnes IB Sattar N. Cardiometabolic comorbidities in RA and PsA: lessons learned and future directions. Nat Rev Rheumatol 2019;15(8):461–74. 31292564 10.1038/s41584-019-0256-0

[4] Gupta S Syrimi Z Hughes DM Zhao SS. Comorbidities in psoriatic arthritis: a systematic review and meta-analysis. Rheumatol Int 2021;41(2):275–84. 33423070 10.1007/s00296-020-04775-2 PMC7835184

[5] Veale DJ Fearon U. The pathogenesis of psoriatic arthritis. Lancet 2018;391(10136):2273–84. 29893226 10.1016/S0140-6736(18)30830-4

[6] Mease PJ Armstrong AW. Managing patients with psoriatic disease: the diagnosis and pharmacologic treatment of psoriatic arthritis in patients with psoriasis. Drugs 2014;74(4):423–41. 24566842 10.1007/s40265-014-0191-y PMC3958815

[7] Gossec L Baraliakos X Kerschbaumer A de Wit M McInnes I Dougados M EULAR recommendations for the management of psoriatic arthritis with pharmacological therapies: 2019 update. Ann Rheum Dis 2020;79(6):700–12. 32434812 10.1136/annrheumdis-2020-217159 PMC7286048

[8] Singh JA Guyatt G Ogdie A Gladman DD Deal C Deodhar A Special Article: 2018 American College of Rheumatology/National Psoriasis Foundation Guideline for the Treatment of Psoriatic Arthritis. Arthritis Rheumatol 2019;71(1):5–32. 30499246 10.1002/art.40726 PMC8218333

[9] Coates LC Kavanaugh A Mease PJ Soriano ER Laura Acosta-Felquer M Armstrong AW Group for Research and Assessment of Psoriasis and Psoriatic Arthritis 2015 Treatment Recommendations for Psoriatic Arthritis. Arthritis Rheumatol 2016;68(5):1060–71. 26749174 10.1002/art.39573

[10] Simons N Degboe Y Barnetche T Cantagrel A Ruyssen-Witrand A Constantin A. Biological DMARD efficacy in psoriatic arthritis: a systematic literature review and meta-analysis on articular, enthesitis, dactylitis, skin and functional outcomes. Clin Exp Rheumatol 2020;38(3):508–15. 31969228

[11] McInnes IB Behrens F Mease PJ Kavanaugh A Ritchlin C Nash P Secukinumab versus adalimumab for treatment of active psoriatic arthritis (EXCEED): a double-blind, parallel-group, randomised, active-controlled, phase 3b trial. Lancet 2020;395(10235):1496–505. 32386593 10.1016/S0140-6736(20)30564-X

[12] Mease PJ van der Heijde D Ritchlin CT Okada M Cuchacovich RS Shuler CL Ixekizumab, an interleukin-17A specific monoclonal antibody, for the treatment of biologic-naive patients with active psoriatic arthritis: results from the 24-week randomised, double-blind, placebo-controlled and active (adalimumab)-controlled period of the phase III trial SPIRIT-P1. Ann Rheum Dis 2017;76(1):79–87. 27553214 10.1136/annrheumdis-2016-209709 PMC5264219

[13] Yang K Oak ASW Elewski BE. Use of IL-23 Inhibitors for the Treatment of Plaque Psoriasis and Psoriatic Arthritis: A Comprehensive Review. Am J Clin Dermatol 2021;22(2):173–92. 33301128 10.1007/s40257-020-00578-0 PMC7727454

[14] Araujo EG Englbrecht M Hoepken S Finzel S Kampylafka E Kleyer A Effects of ustekinumab versus tumor necrosis factor inhibition on enthesitis: Results from the enthesial clearance in psoriatic arthritis (ECLIPSA) study. Semin Arthritis Rheum 2019;48(4):632–7. 30037432 10.1016/j.semarthrit.2018.05.011

[15] Mourad A Gniadecki R. Treatment of Dactylitis and Enthesitis in Psoriatic Arthritis with Biologic Agents: A Systematic Review and Metaanalysis. J Rheumatol 2020;47(1):59–65. 30824641 10.3899/jrheum.180797

[16] Gladman DD Orbai AM Gomez-Reino J Chang-Douglass S Leoncini E Burton HE Network Meta-Analysis of Tofacitinib, Biologic Disease-Modifying Antirheumatic Drugs, and Apremilast for the Treatment of Psoriatic Arthritis. Curr Ther Res Clin Exp 2020;93:100601. 32983284 10.1016/j.curtheres.2020.100601 PMC7494680

[17] Mease PJ Rahman P Gottlieb AB Kollmeier AP Hsia EC Xu XL Guselkumab in biologic-naive patients with active psoriatic arthritis (DISCOVER-2): a double-blind, randomised, placebo-controlled phase 3 trial. Lancet. 2020;395(10230):1126–36. 32178766 10.1016/S0140-6736(20)30263-4

[18] Deodhar A Helliwell PS Boehncke WH Kollmeier AP Hsia EC Subramanian RA Guselkumab in patients with active psoriatic arthritis who were biologic-naive or had previously received TNFalpha inhibitor treatment (DISCOVER-1): a double-blind, randomised, placebo-controlled phase 3 trial. Lancet 2020;395(10230):1115–25. 32178765 10.1016/S0140-6736(20)30265-8

[19] Baraliakos X Gossec L Pournara E Jeka S Mera-Varela A D’Angelo S Secukinumab in patients with psoriatic arthritis and axial manifestations: results from the double-blind, randomised, phase 3 MAXIMISE trial. Ann Rheum Dis 2020 2021 May;80(5):582–90. 33334727 10.1136/annrheumdis-2020-218808 PMC8053347

[20] Lubrano E Spadaro A Marchesoni A Olivieri I Scarpa R D’Angelo S The effectiveness of a biologic agent on axial manifestations of psoriatic arthritis. A twelve months observational study in a group of patients treated with etanercept. Clin Exp Rheumatol 2011;29(1):80–4. 21345296

[21] Sieper J Poddubnyy D Miossec P. The IL-23-IL-17 pathway as a therapeutic target in axial spondyloarthritis. Nat Rev Rheumatol 2019;15(12):747–57. 31551538 10.1038/s41584-019-0294-7

[22] van der Heijde D Deodhar A Wei JC Drescher E Fleishaker D Hendrikx T Tofacitinib in patients with ankylosing spondylitis: a phase II, 16-week, randomised, placebo-controlled, dose-ranging study. Ann Rheum Dis 2017;76(8):1340–7. 28130206 10.1136/annrheumdis-2016-210322 PMC5738601

[23] Whitlock SM Enos CW Armstrong AW Gottlieb A Langley RG Lebwohl M Management of psoriasis in patients with inflammatory bowel disease: From the Medical Board of the National Psoriasis Foundation. J Am Acad Dermatol 2018;78(2):383–94. 29332708 10.1016/j.jaad.2017.06.043

[24] Sbidian E Chaimani A Afach S Doney L Dressler C Hua C Systemic pharmacological treatments for chronic plaque psoriasis: a network meta-analysis. Cochrane Database Syst Rev 2020;1:CD011535. 31917873 10.1002/14651858.CD011535.pub3 PMC6956468

[25] Ash Z Gaujoux-Viala C Gossec L Hensor EM FitzGerald O Winthrop K A systematic literature review of drug therapies for the treatment of psoriatic arthritis: current evidence and meta-analysis informing the EULAR recommendations for the management of psoriatic arthritis. Ann Rheum Dis 2012;71(3):319–26. 21803753 10.1136/ard.2011.150995

[26] Griffiths CE Strober BE van de Kerkhof P Ho V Fidelus-Gort R Yeilding N Comparison of ustekinumab and etanercept for moderate-to-severe psoriasis. N Engl J Med 2010;362(2):118–28. 20071701 10.1056/NEJMoa0810652

[27] Langley RG Elewski BE Lebwohl M Reich K Griffiths CE Papp K Secukinumab in plaque psoriasis--results of two phase 3 trials. N Engl J Med 2014;371(4):326–38. 25007392 10.1056/NEJMoa1314258

[28] Griffiths CE Reich K Lebwohl M van de Kerkhof P Paul C Menter A Comparison of ixekizumab with etanercept or placebo in moderate-to-severe psoriasis (UNCOVER-2 and UNCOVER-3): results from two phase 3 randomised trials. Lancet 2015;386(9993):541–51. 26072109 10.1016/S0140-6736(15)60125-8

[29] Reich K Armstrong AW Foley P Song M Wasfi Y Randazzo B Efficacy and safety of guselkumab, an anti-interleukin-23 monoclonal antibody, compared with adalimumab for the treatment of patients with moderate to severe psoriasis with randomized withdrawal and retreatment: Results from the phase III, double-blind, placebo- and active comparator-controlled VOYAGE 2 trial. J Am Acad Dermatol 2017;76(3):418–31. 28057361 10.1016/j.jaad.2016.11.042

[30] Schett G Lories RJ D’Agostino MA Elewaut D Kirkham B Soriano ER Enthesitis: from pathophysiology to treatment. Nat Rev Rheumatol 2017;13(12):731–41. 29158573 10.1038/nrrheum.2017.188

[31] Mease P Hall S FitzGerald O van der Heijde D Merola JF Avila-Zapata F Tofacitinib or Adalimumab versus Placebo for Psoriatic Arthritis. N Engl J Med 2017;377(16):1537–50. 29045212 10.1056/NEJMoa1615975

[32] Mease PJ Gladman DD Deodhar A McGonagle DG Nash P Boehncke WH Impact of guselkumab, an interleukin-23 p19 subunit inhibitor, on enthesitis and dactylitis in patients with moderate to severe psoriatic arthritis: results from a randomised, placebo-controlled, phase II study. RMD Open 2020;6(2). 10.1136/rmdopen-2020-001217 PMC7425189 32665433

[33] McGonagle D Tan AL Watad A Helliwell P. Pathophysiology, assessment and treatment of psoriatic dactylitis. Nat Rev Rheumatol 2019;15(2):113–22. 30610219 10.1038/s41584-018-0147-9

[34] Vieira-Sousa E Alves P Rodrigues AM Teixeira F Tavares-Costa J Bernardo A GO-DACT: a phase 3b randomised, double-blind, placebo-controlled trial of GOlimumab plus methotrexate (MTX) versus placebo plus MTX in improving DACTylitis in MTX-naive patients with psoriatic arthritis. Ann Rheum Dis 2020;79(4):490–8. 32193187 10.1136/annrheumdis-2019-216500 PMC7147178

[35] Kavanaugh A Puig L Gottlieb AB Ritchlin C You Y Li S Efficacy and safety of ustekinumab in psoriatic arthritis patients with peripheral arthritis and physician-reported spondylitis: post-hoc analyses from two phase III, multicentre, double-blind, placebo-controlled studies (PSUMMIT-1/PSUMMIT-2). Ann Rheum Dis 2016;75(11):1984–8. 27098404 10.1136/annrheumdis-2015-209068

[36] Sobolewski P Walecka I Dopytalska K. Nail involvement in psoriatic arthritis. Reumatologia 2017;55(3):131–5. 28769136 10.5114/reum.2017.68912 PMC5534507

[37] Reich K Nestle FO Papp K Ortonne JP Evans R Guzzo C Infliximab induction and maintenance therapy for moderate-to-severe psoriasis: a phase III, multicentre, double-blind trial. Lancet 2005;366(9494):1367–74. 16226614 10.1016/S0140-6736(05)67566-6

[38] Elyoussfi S Thomas BJ Ciurtin C. Tailored treatment options for patients with psoriatic arthritis and psoriasis: review of established and new biologic and small molecule therapies. Rheumatol Int 2016;36(5):603–12. 26892034 10.1007/s00296-016-3436-0 PMC4839046

[39] Kavanaugh A McInnes I Mease P Krueger GG Gladman D Gomez-Reino J Golimumab, a new human tumor necrosis factor alpha antibody, administered every four weeks as a subcutaneous injection in psoriatic arthritis: Twenty-four-week efficacy and safety results of a randomized, placebo-controlled study. Arthritis Rheum 2009;60(4):976–86. 19333944 10.1002/art.24403

[40] Mease PJ Fleischmann R Deodhar AA Wollenhaupt J Khraishi M Kielar D Effect of certolizumab pegol on signs and symptoms in patients with psoriatic arthritis: 24-week results of a Phase 3 double-blind randomised placebo-controlled study (RAPID-PsA). Ann Rheum Dis 2014;73(1):48–55. 23942868 10.1136/annrheumdis-2013-203696 PMC3888622

[41] Rich P Bourcier M Sofen H Fakharzadeh S Wasfi Y Wang Y Ustekinumab improves nail disease in patients with moderate-to-severe psoriasis: results from PHOENIX 1. Br J Dermatol 2014;170(2):398–407. 24117389 10.1111/bjd.12632

[42] Reich K Sullivan J Arenberger P Jazayeri S Mrowietz U Augustin M Secukinumab shows high and sustained efficacy in nail psoriasis: 2.5-year results from the randomized placebo-controlled TRANSFIGURE study. Br J Dermatol 2021;184(3):425–36. 32479641 10.1111/bjd.19262

[43] Foley P Gordon K Griffiths CEM Wasfi Y Randazzo B Song M Efficacy of Guselkumab Compared With Adalimumab and Placebo for Psoriasis in Specific Body Regions: A Secondary Analysis of 2 Randomized Clinical Trials. JAMA Dermatol 2018;154(6):676–83. 29799960 10.1001/jamadermatol.2018.0793 PMC6145649

[44] Merola JF Elewski B Tatulych S Lan S Tallman A Kaur M. Efficacy of tofacitinib for the treatment of nail psoriasis: Two 52-week, randomized, controlled phase 3 studies in patients with moderate-to-severe plaque psoriasis. J Am Acad Dermatol 2017;77(1):79–87e1. 28396102 10.1016/j.jaad.2017.01.053

[45] Sanchez-Bilbao L Martinez-Lopez D Palmou-Fontana N Armesto S González-Gay MA Blanco R. Ab0829 Inflammatory Bowel Disease in Psoriatic Arthritis. Study of 306 Patients from a Single Universitary Center. Prevalence, Clinical Features and Relationship to Biologic Therapy. Ann Rheum Dis 2020;79 (Suppl 1):1719.2-.

[46] Scarpa R Manguso F D’Arienzo A D’Armiento FP Astarita C Mazzacca G Microscopic inflammatory changes in colon of patients with both active psoriasis and psoriatic arthritis without bowel symptoms. J Rheumatol 2000;27(5):1241–6. 10813294

[47] Pugliese D Daperno M Fiorino G Savarino E Mosso E Biancone L Real-life effectiveness of ustekinumab in inflammatory bowel disease patients with concomitant psoriasis or psoriatic arthritis: An IG-IBD study. Dig Liver Dis 2019;51(7):972–7. 30992173 10.1016/j.dld.2019.03.007

[48] So A Inman RD. An overview of biologic disease-modifying antirheumatic drugs in axial spondyloarthritis and psoriatic arthritis. Best Pract Res Clin Rheumatol 2018;32(3):453–71. 31171315 10.1016/j.berh.2018.12.002

[49] Schreiber S Colombel JF Feagan BG Reich K Deodhar AA McInnes IB Incidence rates of inflammatory bowel disease in patients with psoriasis, psoriatic arthritis and ankylosing spondylitis treated with secukinumab: a retrospective analysis of pooled data from 21 clinical trials. Ann Rheum Dis 2019;78(4):473–9. 30674475 10.1136/annrheumdis-2018-214273 PMC6530077

[50] Panes J Sandborn WJ Schreiber S Sands BE Vermeire S D’Haens G Tofacitinib for induction and maintenance therapy of Crohn’s disease: results of two phase IIb randomised placebo-controlled trials. Gut 2017;66(6):1049–59. 28209624 10.1136/gutjnl-2016-312735 PMC5532457

[51] Perez-Chada LM Merola JF. Comorbidities associated with psoriatic arthritis: Review and update. Clin Immunol 2020;214:108397. 32229290 10.1016/j.clim.2020.108397

[52] Arepalli S Rosenbaum JT. The use of biologics for uveitis associated with spondyloarthritis. Curr Opin Rheumatol 2019;31(4):349–54. 31107288 10.1097/BOR.0000000000000619

[53] Bruner M Dige A Loft AG Laurberg TB Agnholt JS Clemmensen K Spondylitis-psoriasis-enthesitis-enterocolitis-dactylitis-uveitis-peripheral synovitis (SPEED-UP) treatment. Autoimmun Rev 2021;20(2):102731. 33326852 10.1016/j.autrev.2020.102731

[54] Dick AD Tugal-Tutkun I Foster S Zierhut M Melissa Liew SH Bezlyak V Secukinumab in the treatment of noninfectious uveitis: results of three randomized, controlled clinical trials. Ophthalmology 2013;120(4):777–87. 23290985 10.1016/j.ophtha.2012.09.040

[55] Scher JU Ogdie A Merola JF Ritchlin C. Preventing psoriatic arthritis: focusing on patients with psoriasis at increased risk of transition. Nat Rev Rheumatol 2019;15(3):153–66. 30742092 10.1038/s41584-019-0175-0

[56] Patsalos O Dalton B Leppanen J Ibrahim MAA Himmerich H. Impact of TNF-alpha Inhibitors on Body Weight and BMI: A Systematic Review and Meta-Analysis. Front Pharmacol 2020;11:481. 32351392 10.3389/fphar.2020.00481 PMC7174757

[57] Toussirot E Mourot L Dehecq B Wendling D Grandclement E Dumoulin G TNFalpha blockade for inflammatory rheumatic diseases is associated with a significant gain in android fat mass and has varying effects on adipokines: a 2-year prospective study. Eur J Nutr 2014;53(3):951–61. 24173963 10.1007/s00394-013-0599-2

[58] di Minno MN Peluso R Iervolino S Lupoli R Russolillo A Scarpa R Obesity and the prediction of minimal disease activity: a prospective study in psoriatic arthritis. Arthritis Care Res (Hoboken) 2013;65(1):141–7. 22514189 10.1002/acr.21711

[59] Eder L Thavaneswaran A Chandran V Cook RJ Gladman DD. Obesity is associated with a lower probability of achieving sustained minimal disease activity state among patients with psoriatic arthritis. Ann Rheum Dis 2015;74(5):813–7. 24431392 10.1136/annrheumdis-2013-204448

[60] Costa L Caso F Ramonda R Del Puente A Cantarini L Darda MA Metabolic syndrome and its relationship with the achievement of minimal disease activity state in psoriatic arthritis patients: an observational study. Immunol Res 2015;61(1–2):147–53. 25395342 10.1007/s12026-014-8595-z

[61] Ogdie A Palmer JL Greenberg J Curtis JR Harrold LR Solomon DH Predictors of Achieving Remission among Patients with Psoriatic Arthritis Initiating a Tumor Necrosis Factor Inhibitor. J Rheumatol 2019;46(5):475–82. 30647182 10.3899/jrheum.171034

[62] Hojgaard P Glintborg B Kristensen LE Gudbjornsson B Love TJ Dreyer L. The influence of obesity on response to tumour necrosis factor-alpha inhibitors in psoriatic arthritis: results from the DANBIO and ICEBIO registries. Rheumatology (Oxford) 2016;55(12):2191–9. 27651526 10.1093/rheumatology/kew326

[63] Chehimi M Vidal H Eljaafari A. Pathogenic Role of IL-17-Producing Immune Cells in Obesity, and Related Inflammatory Diseases. J Clin Med 2017;6(7). 10.3390/jcm6070068 PMC5532576 28708082

[64] Pantano I Iacono D Favalli EG Scalise G Costa L Caso F Secukinumab efficacy in patients with PsA is not dependent on patients’ body mass index. Ann Rheum Dis 2020. 10.1136/annrheumdis-2020-217251 32169970

[65] Notario J Deza G Vilarrasa E Valenti F Munoz C Mollet J Treatment of patients with plaque psoriasis with secukinumab in a real-life setting: a 52-week, multicenter, retrospective study in Spain. J Dermatolog Treat 2019;30(5):424–9. 30244618 10.1080/09546634.2018.1528000

[66] Dal Bello G Gisondi P Idolazzi L Girolomoni G. Psoriatic Arthritis and Diabetes Mellitus: A Narrative Review. Rheumatol Ther 2020;7(2):271–85. 32306243 10.1007/s40744-020-00206-7 PMC7211212

[67] Zhang C Xiao C Wang P Xu W Zhang A Li Q The alteration of Th1/Th2/Th17/Treg paradigm in patients with type 2 diabetes mellitus: Relationship with diabetic nephropathy. Hum Immunol 2014;75(4):289–96. 24530745 10.1016/j.humimm.2014.02.007

[68] da Silva BS Bonfa E de Moraes JC Saad CG Ribeiro AC Goncalves CR Effects of anti-TNF therapy on glucose metabolism in patients with ankylosing spondylitis, psoriatic arthritis or juvenile idiopathic arthritis. Biologicals 2010;38(5):567–9. 20638299 10.1016/j.biologicals.2010.05.003

[69] Mantravadi S George M Brensinger C Du M Baker JF Ogdie A. Impact of tumor necrosis factor inhibitors and methotrexate on diabetes mellitus among patients with inflammatory arthritis. BMC Rheumatol 2020;4:39. 32905192 10.1186/s41927-020-00138-3 PMC7466800

[70] Egeberg A Wu JJ Korman N Solomon JA Goldblum O Zhao F Ixekizumab treatment shows a neutral impact on cardiovascular parameters in patients with moderate-to-severe plaque psoriasis: Results from UNCOVER-1, UNCOVER-2, and UNCOVER-3. J Am Acad Dermatol 2018;79(1):104–9e8. 29548945 10.1016/j.jaad.2018.02.074

[71] Frieder J Kivelevitch D Menter A. Secukinumab: a review of the anti-IL-17A biologic for the treatment of psoriasis. Ther Adv Chronic Dis 2018;9(1):5–21. 29344327 10.1177/2040622317738910 PMC5761942

[72] Gerdes S Pinter A Papavassilis C Reinhardt M. Effects of secukinumab on metabolic and liver parameters in plaque psoriasis patients. J Eur Acad Dermatol Venereol 2020;34(3):533–41. 31599476 10.1111/jdv.16004 PMC7065121

[73] O’Rielly DD Rahman P. A review of ixekizumab in the treatment of psoriatic arthritis. Expert Rev Clin Immunol 2018;14(12):993–1002. 30360663 10.1080/1744666X.2018.1540931

[74] Ritchlin CT Giles JT Ogdie A Gomez-Reino JJ Helliwell P Young P Tofacitinib in Patients With Psoriatic Arthritis and Metabolic Syndrome: A Post hoc Analysis of Phase 3 Studies. ACR Open Rheumatol 2020;2(10):543–54. 32910531 10.1002/acr2.11166 PMC7571390

[75] Sattar N Forrest E Preiss D. Non-alcoholic fatty liver disease. BMJ 2014;349:g4596. 25239614 10.1136/bmj.g4596 PMC4168663

[76] Candia R Ruiz A Torres-Robles R Chavez-Tapia N Mendez-Sanchez N Arrese M. Risk of non-alcoholic fatty liver disease in patients with psoriasis: a systematic review and meta-analysis. J Eur Acad Dermatol Venereol 2015;29(4):656–62. 25418531 10.1111/jdv.12847

[77] Ortolan A Lorenzin M Tadiotto G Russo FP Oliviero F Felicetti M Metabolic syndrome, non-alcoholic fatty liver disease and liver stiffness in psoriatic arthritis and psoriasis patients. Clin Rheumatol 2019;38(10):2843–50. 31254236 10.1007/s10067-019-04646-7

[78] Seitz M Reichenbach S Moller B Zwahlen M Villiger PM Dufour JF. Hepatoprotective effect of tumour necrosis factor alpha blockade in psoriatic arthritis: a cross-sectional study. Ann Rheum Dis 2010;69(6):1148–50. 19854710 10.1136/ard.2009.116194

[79] Tang KT Dufour JF Chen PH Hernaez R Hutfless S. Antitumour necrosis factor-alpha agents and development of new-onset cirrhosis or non-alcoholic fatty liver disease: a retrospective cohort. BMJ Open Gastroenterol 2020;7(1):e000349. 10.1136/bmjgast-2019-000349 PMC7199652 32377366

[80] Tang Y Bian Z Zhao L Liu Y Liang S Wang Q Interleukin-17 exacerbates hepatic steatosis and inflammation in non-alcoholic fatty liver disease. Clin Exp Immunol 2011;166(2):281–90. 21985374 10.1111/j.1365-2249.2011.04471.x PMC3219903

[81] Jamnitski A Symmons D Peters MJ Sattar N McInnes I Nurmohamed MT. Cardiovascular comorbidities in patients with psoriatic arthritis: a systematic review. Ann Rheum Dis 2013;72(2):211–6. 22532629 10.1136/annrheumdis-2011-201194

[82] Myasoedova E Crowson CS Kremers HM Roger VL Fitz-Gibbon PD Therneau TM Lipid paradox in rheumatoid arthritis: the impact of serum lipid measures and systemic inflammation on the risk of cardiovascular disease. Ann Rheum Dis 2011;70(3):482–7. 21216812 10.1136/ard.2010.135871 PMC3058921

[83] Agca R Heslinga M Kneepkens EL van Dongen C Nurmohamed MT. The Effects of 5-year Etanercept Therapy on Cardiovascular Risk Factors in Patients with Psoriatic Arthritis. J Rheumatol 2017;44(9):1362–8. 28572461 10.3899/jrheum.161418

[84] Fragoulis GE Panayotidis I Nikiphorou E. Cardiovascular Risk in Rheumatoid Arthritis and Mechanistic Links: From Pathophysiology to Treatment. Curr Vasc Pharmacol 2020;18(5):431–46. 31258091 10.2174/1570161117666190619143842

[85] Polachek A Touma Z Anderson M Eder L. Risk of Cardiovascular Morbidity in Patients With Psoriatic Arthritis: A Meta-Analysis of Observational Studies. Arthritis Care Res (Hoboken) 2017;69(1):67–74. 27111228 10.1002/acr.22926

[86] Hot A Lenief V Miossec P. Combination of IL-17 and TNFalpha induces a pro-inflammatory, pro-coagulant and pro-thrombotic phenotype in human endothelial cells. Ann Rheum Dis 2012;71(5):768–76. 22258491 10.1136/annrheumdis-2011-200468

[87] Di Minno MN Iervolino S Peluso R Scarpa R Di Minno G Rsg. Ca Carotid intima-media thickness in psoriatic arthritis: differences between tumor necrosis factor-alpha blockers and traditional disease-modifying antirheumatic drugs. Arterioscler Thromb Vasc Biol 2011;31(3):705–12. 21212403 10.1161/ATVBAHA.110.214585

[88] Eder L Joshi AA Dey AK Cook R Siegel EL Gladman DD Association of Tumor Necrosis Factor Inhibitor Treatment With Reduced Indices of Subclinical Atherosclerosis in Patients With Psoriatic Disease. Arthritis Rheumatol 2018;70(3):408–16. 29088580 10.1002/art.40366

[89] Roubille C Richer V Starnino T McCourt C McFarlane A Fleming P The effects of tumour necrosis factor inhibitors, methotrexate, non-steroidal anti-inflammatory drugs and corticosteroids on cardiovascular events in rheumatoid arthritis, psoriasis and psoriatic arthritis: a systematic review and meta-analysis. Ann Rheum Dis 2015;74(3):480–9. 25561362 10.1136/annrheumdis-2014-206624 PMC4345910

[90] Yang ZS Lin NN Li L Li Y. The Effect of TNF Inhibitors on Cardiovascular Events in Psoriasis and Psoriatic Arthritis: an Updated Meta-Analysis. Clin Rev Allergy Immunol 2016;51(2):240–7. 27300248 10.1007/s12016-016-8560-9

[91] Lee MP Desai RJ Jin Y Brill G Ogdie A Kim SC. Association of Ustekinumab vs TNF Inhibitor Therapy With Risk of Atrial Fibrillation and Cardiovascular Events in Patients With Psoriasis or Psoriatic Arthritis. JAMA Dermatol 2019;155(6):700–7. 30916734 10.1001/jamadermatol.2019.0001 PMC6563547

[92] Nash P Coates LC Kivitz AJ Mease PJ Gladman DD Covarrubias-Cobos JA Safety and Efficacy of Tofacitinib in Patients with Active Psoriatic Arthritis: Interim Analysis of OPAL Balance, an Open-Label, Long-Term Extension Study. Rheumatol Ther 2020;7(3):553–80. 32506317 10.1007/s40744-020-00209-4 PMC7410915

[93] Michelsen B Kristianslund EK Sexton J Hammer HB Fagerli KM Lie E Do depression and anxiety reduce the likelihood of remission in rheumatoid arthritis and psoriatic arthritis? Data from the prospective multicentre NOR-DMARD study. Ann Rheum Dis 2017;76(11):1906–10. 28733473 10.1136/annrheumdis-2017-211284

[94] Kappelmann N Lewis G Dantzer R Jones PB Khandaker GM. Antidepressant activity of anti-cytokine treatment: a systematic review and meta-analysis of clinical trials of chronic inflammatory conditions. Mol Psychiatry 2018;23(2):335–43. 27752078 10.1038/mp.2016.167 PMC5794896

[95] Wittenberg GM Stylianou A Zhang Y Sun Y Gupta A Jagannatha PS Effects of immunomodulatory drugs on depressive symptoms: A mega-analysis of randomized, placebo-controlled clinical trials in inflammatory disorders. Mol Psychiatry 2020;25(6):1275–85. 31427751 10.1038/s41380-019-0471-8 PMC7244402

[96] Sepriano A Kerschbaumer A Smolen JS van der Heijde D Dougados M van Vollenhoven R Safety of synthetic and biological DMARDs: a systematic literature review informing the 2019 update of the EULAR recommendations for the management of rheumatoid arthritis. Ann Rheum Dis 2020;79(6):760–70. 32033941 10.1136/annrheumdis-2019-216653

[97] EMA confirms Xeljanz to be used with caution in patients at high risk of blood clots [press release]. 31 January 2020.

[98] Winthrop KL Citera G Gold D Henrohn D Connell CA Shapiro AB Age-based (<65 vs >/=65 years) incidence of infections and serious infections with tofacitinib versus biological DMARDs in rheumatoid arthritis clinical trials and the US Corrona RA registry. Ann Rheum Dis 2021;80(1):134–6. 33046447 10.1136/annrheumdis-2020-218992 PMC7788057

[99] Arkema EV Jonsson J Baecklund E Bruchfeld J Feltelius N Askling J Are patients with rheumatoid arthritis still at an increased risk of tuberculosis and what is the role of biological treatments? Ann Rheum Dis 2015;74(6):1212–7. 24608401 10.1136/annrheumdis-2013-204960

[100] Minozzi S Bonovas S Lytras T Pecoraro V Gonzalez-Lorenzo M Bastiampillai AJ Risk of infections using anti-TNF agents in rheumatoid arthritis, psoriatic arthritis, and ankylosing spondylitis: a systematic review and meta-analysis. Expert Opin Drug Saf 2016;15(sup1):11–34. 27924643 10.1080/14740338.2016.1240783

[101] Seong SS Choi CB Woo JH Bae KW Joung CL Uhm WS Incidence of tuberculosis in Korean patients with rheumatoid arthritis (RA): effects of RA itself and of tumor necrosis factor blockers. J Rheumatol 2007;34(4):706–11. 17309133

[102] Bekker LG Freeman S Murray PJ Ryffel B Kaplan G. TNF-alpha controls intracellular mycobacterial growth by both inducible nitric oxide synthase-dependent and inducible nitric oxide synthase-independent pathways. J Immunol 2001;166(11):6728–34. 11359829 10.4049/jimmunol.166.11.6728

[103] Roach DR Bean AG Demangel C France MP Briscoe H Britton WJ. TNF regulates chemokine induction essential for cell recruitment, granuloma formation, and clearance of mycobacterial infection. J Immunol 2002;168(9):4620–7. 11971010 10.4049/jimmunol.168.9.4620

[104] Tubach F Salmon D Ravaud P Allanore Y Goupille P Breban M Risk of tuberculosis is higher with anti-tumor necrosis factor monoclonal antibody therapy than with soluble tumor necrosis factor receptor therapy: The three-year prospective French Research Axed on Tolerance of Biotherapies registry. Arthritis Rheum 2009;60(7):1884–94. 19565495 10.1002/art.24632 PMC2921546

[105] Domingo-Gonzalez R Prince O Cooper A Khader SA. Cytokines and Chemokines in Mycobacterium tuberculosis Infection. Microbiol Spectr 2016;4(5). 10.1128/microbiolspec.TBTB2-0018-2016 PMC5205539 27763255

[106] Deodhar A Mease PJ McInnes IB Baraliakos X Reich K Blauvelt A Long-term safety of secukinumab in patients with moderate-to-severe plaque psoriasis, psoriatic arthritis, and ankylosing spondylitis: integrated pooled clinical trial and post-marketing surveillance data. Arthritis Res Ther 2019;21(1):111. 31046809 10.1186/s13075-019-1882-2 PMC6498580

[107] Romiti R Valenzuela F Chouela EN Xu W Pangallo B Moriarty SR Prevalence and outcome of latent tuberculosis in patients receiving ixekizumab: integrated safety analysis from 11 clinical trials of patients with plaque psoriasis. Br J Dermatol 2019;181(1):202–3. 30609008 10.1111/bjd.17604 PMC6900236

[108] Wu CY Chiu HY Tsai TF. The seroconversion rate of QuantiFERONTB Gold In-Tube test in psoriatic patients receiving secukinumab and ixekizumab, the anti-interleukin-17A monoclonal antibodies. PLoS One 2019;14(12):e0225112. 31881026 10.1371/journal.pone.0225112 PMC6934285

[109] Mata-Espinosa DA Francisco-Cruz A Marquina-Castillo B Barrios-Payan J Ramos-Espinosa O Bini EI Immunotherapeutic effects of recombinant adenovirus encoding interleukin 12 in experimental pulmonary tuberculosis. Scand J Immunol 2019;89(3):e12743. 30548932 10.1111/sji.12743

[110] McInnes IB Kavanaugh A Gottlieb AB Puig L Rahman P Ritchlin C Efficacy and safety of ustekinumab in patients with active psoriatic arthritis: 1 year results of the phase 3, multi-centre, double-blind, placebo-controlled PSUMMIT 1 trial. Lancet 2013;382(9894):780–9. 23769296 10.1016/S0140-6736(13)60594-2

[111] Ritchlin C Rahman P Kavanaugh A McInnes IB Puig L Li S Efficacy and safety of the anti-IL-12/23 p40 monoclonal antibody, ustekinumab, in patients with active psoriatic arthritis despite conventional non-biological and biological anti-tumour necrosis factor therapy: 6-month and 1-year results of the phase 3, multicentre, double-blind, placebo-controlled, randomised PSUMMIT 2 trial. Ann Rheum Dis 2014;73(6):990–9. 24482301 10.1136/annrheumdis-2013-204655 PMC4033144

[112] Deodhar A Gottlieb AB Boehncke WH Dong B Wang Y Zhuang Y Efficacy and safety of guselkumab in patients with active psoriatic arthritis: a randomised, double-blind, placebo-controlled, phase 2 study. Lancet 2018;391(10136):2213–24. 29893222 10.1016/S0140-6736(18)30952-8

[113] Fragoulis GE McInnes IB Siebert S. JAK-inhibitors. New players in the field of immune-mediated diseases, beyond rheumatoid arthritis. Rheumatology (Oxford) 2019;58(Suppl 1):i43–i54. 30806709 10.1093/rheumatology/key276 PMC6390879

[114] Boisson-Dupuis S Ramirez-Alejo N Li Z Patin E Rao G Kerner G Tuberculosis and impaired IL-23-dependent IFN-gamma immunity in humans homozygous for a common TYK2 missense variant. Sci Immunol 2018;3(30). 10.1126/sciimmunol.aau8714 PMC6341984 30578352

[115] Winthrop KL Park SH Gul A Cardiel MH Gomez-Reino JJ Tanaka Y Tuberculosis and other opportunistic infections in tofacitinib-treated patients with rheumatoid arthritis. Ann Rheum Dis 2016;75(6):1133–8. 26318385 10.1136/annrheumdis-2015-207319 PMC4893093

[116] Gladman D Rigby W Azevedo VF Behrens F Blanco R Kaszuba A Tofacitinib for Psoriatic Arthritis in Patients with an Inadequate Response to TNF Inhibitors. N Engl J Med 2017;377(16):1525–36. 29045207 10.1056/NEJMoa1615977

[117] Nash P Kerschbaumer A Dorner T Dougados M Fleischmann RM Geissler K Points to consider for the treatment of immune-mediated inflammatory diseases with Janus kinase inhibitors: a consensus statement. Ann Rheum Dis 2021;80(1):71–87. 33158881 10.1136/annrheumdis-2020-218398 PMC7788060

[118] Sunzini F McInnes I Siebert S. JAK inhibitors and infections risk: focus on herpes zoster. Ther Adv Musculoskelet Dis. 2020;12:1759720X20936059. 10.1177/1759720X20936059 PMC7328488 32655703

[119] Curtis JR Xie F Yang S Bernatsky S Chen L Yun H Risk for Herpes Zoster in Tofacitinib-Treated Rheumatoid Arthritis Patients With and Without Concomitant Methotrexate and Glucocorticoids. Arthritis Care Res (Hoboken) 2019;71(9):1249–54. 30295428 10.1002/acr.23769

[120] Winthrop KL Curtis JR Lindsey S Tanaka Y Yamaoka K Valdez H Herpes Zoster and Tofacitinib: Clinical Outcomes and the Risk of Concomitant Therapy. Arthritis Rheumatol 2017;69(10):1960–8. 28845604 10.1002/art.40189 PMC5656820

[121] Winthrop KL Yamanaka H Valdez H Mortensen E Chew R Krishnaswami S Herpes zoster and tofacitinib therapy in patients with rheumatoid arthritis. Arthritis Rheumatol 2014;66(10):2675–84. 24943354 10.1002/art.38745 PMC4285807

[122] Kivitz AJ Cohen S Keystone E van Vollenhoven RF Haraoui B Kaine J A pooled analysis of the safety of tofacitinib as monotherapy or in combination with background conventional synthetic disease-modifying antirheumatic drugs in a Phase 3 rheumatoid arthritis population. Semin Arthritis Rheum 2018;48(3):406–15. 30177460 10.1016/j.semarthrit.2018.07.006

[123] Braun J Baraliakos X Deodhar A Poddubnyy D Emery P Delicha EM Secukinumab shows sustained efficacy and low structural progression in ankylosing spondylitis: 4-year results from the MEASURE 1 study. Rheumatology (Oxford) 2019;58(5):859–68. 30590813 10.1093/rheumatology/key375 PMC6477523

[124] Puel A Cypowyj S Bustamante J Wright JF Liu L Lim HK Chronic mucocutaneous candidiasis in humans with inborn errors of interleukin-17 immunity. Science 2011;332(6025):65–8. 21350122 10.1126/science.1200439 PMC3070042

[125] Fagerli KM Kearsley-Fleet L Mercer LK Watson K Packham J Symmons DPM Malignancy and mortality rates in patients with severe psoriatic arthritis requiring tumour-necrosis factor alpha inhibition: results from the British Society for Rheumatology Biologics Register. Rheumatology (Oxford) 2019;58(1):80–5. 30137485 10.1093/rheumatology/key241 PMC6293477

[126] Kwan YH Lim KK Fong W Goh H Ng L Haaland B Risk of malignancies in patients with spondyloarthritis treated with biologics compared with those treated with non-biologics: a systematic review and meta-analysis. Ther Adv Musculoskelet Dis 2020;12:1759720X20925696. 10.1177/1759720X20925696 PMC7573508 33149771

[127] Luo X Deng C Fei Y Zhang W Li Y Zhang X Malignancy development risk in psoriatic arthritis patients undergoing treatment: A systematic review and meta-analysis. Semin Arthritis Rheum 2019;48(4):626–31. 29929736 10.1016/j.semarthrit.2018.05.009

[128] Kerschbaumer A Smolen JS Nash P Doerner T Dougados M Fleischmann R Points to consider for the treatment of immune-mediated inflammatory diseases with Janus kinase inhibitors: a systematic literature research. RMD Open 2020;6(3). 10.1136/rmdopen-2020-001374 PMC7856126 33188136

[129] Smolen JS Genovese MC Takeuchi T Hyslop DL Macias WL Rooney T Safety Profile of Baricitinib in Patients with Active Rheumatoid Arthritis with over 2 Years Median Time in Treatment. J Rheumatol 2019;46(1):7–18. 30219772 10.3899/jrheum.171361

[130] Taylor PC Weinblatt ME Burmester GR Rooney TP Witt S Walls CD Cardiovascular Safety During Treatment With Baricitinib in Rheumatoid Arthritis. Arthritis Rheumatol 2019;71(7):1042–55. 30663869 10.1002/art.40841 PMC6618316

[131] Heslinga SC Van Sijl AM De Boer K Van Halm VP Nurmohamed MT. Tumor necrosis factor blocking therapy and congestive heart failure in patients with inflammatory rheumatic disorders: a systematic review. Curr Med Chem 2015;22(16):1892–902. 25666788 10.2174/0929867322666150209160701

